# Aquaporin 1 is located on the intestinal basolateral membrane in *Toxocara canis* and might play a role in drug uptake

**DOI:** 10.1186/s13071-019-3500-1

**Published:** 2019-05-17

**Authors:** Guangxu Ma, Aiyun Jiang, Yongfang Luo, Yongli Luo, Hancheng Huang, Rongqiong Zhou

**Affiliations:** 1 Department of Veterinary Medicine, College of Animal Science, Southwest University, Chongqing, 402460 The People’s Republic of China; 2 Faculty of Veterinary and Agricultural Sciences, The University of Melbourne, Parkville, VIC 3010 Australia

**Keywords:** *Toxocara canis*, Aquaporin 1, Tissue distribution, Reproduction, Drug uptake

## Abstract

**Background:**

Aquaporins (AQPs) are a family of integral membrane channel proteins that facilitate the transport of water and other small solutes across cell membranes. AQPs appear to play crucial roles in parasite survival and represent possible drug targets for novel intervention strategy. In this work, we investigated the tissue distribution and biological roles of an aquaporin *Tc*AQP1 in the neglected parasitic nematode *Toxocara canis*.

**Methods:**

Recombinant C-terminal hydrophilic domain of AQP1 of *T. canis* (r*Tc*AQP1c) and polyclonal antibody against r*Tc*AQP1c were produced to analyse the tissue expression of native *Tc*AQP1 in adult (female and male) worms using an immunohistochemical approach. RNA interference (RNAi), quantitative real-time PCR (qRT-PCR) and nematocidal assays were performed to investigate the functional roles of *Tc*AQP1 in the adult stage of *T. canis*.

**Results:**

Immunofluorescence analysis showed that *Tc*AQP1 was localised predominantly in the epithelial linings of the reproductive tract and basolateral membrane of the intestine in the adult stage (female and male) of *T. canis*, indicating important roles in reproduction, nutrient absorption and/or osmoregulation. Treatment with silencing RNA for 24 h resulted in a significant reduction of *Tc-aqp-1* mRNA level in adult *T. canis*, though no phenotypical change was observed. The efficient gene knockdown compromised the nematocidal activity of albendazole *in vitro*, suggesting the role of *Tc*AQP1 in drug uptake.

**Conclusions:**

The findings of this study provide important information about tissue expression and functional roles of *Tc*AQP1 protein in adult *T. canis*. Understanding the biological functions of this protein in other developmental stages of *T. canis* and related parasitic nematodes would contribute to the discovery of novel diagnostic or anthelmintic targets.

## Background

Transmembrane transport is a fundamental process of the cellular activity. Aquaporin 1, an integral membrane channel protein facilitating the transport of water, was first identified in human erythrocytes [1, 2]. Over the years, more aquaporins (AQPs) have been identified in a number of prokaryotic and eukaryotic organisms [3–8]. Apart from water transport, these proteins have been shown to play roles in the transportation of glycerol, gases (e.g. CO_2_, NH_3_, NO and O_2_), small solutes (e.g. H_2_O_2_ and arsenite) and/or ions (e.g. K^+^ and Cl^−^) across cell membranes [9, 10]. However, studies indicated that these AQPs appear to be structurally and functionally divergent among organisms [11–15].

In parasites, particularly protozoans and platyhelminths, AQPs have been reported to have roles in the regulation of osmotic pressure, nutrient absorption, metabolic product efflux and/or host-parasite interactions [16–19]. Specifically, some AQPs have been proposed to play roles in the drug sensitivity and resistance of parasites, such as *Leishmania* spp., *Trypanosoma brucei* and *Schistosoma mansoni* [20–23]. These findings have been raising interest in the proposition that AQPs might represent novel therapeutic targets for parasitic diseases [24–27]. However, little is known about this area in parasitic nematodes, though selected genes coding for some AQPs have been cloned and analysed [28–30]. A better understanding of this area in parasitic nematodes would contribute to the discovery of novel anthelmintic targets.

*Toxocara canis*, the roundworm commonly found in canids and the main causative agent for human toxocariasis, is a parasitic nematode of public health importance to be investigated [31, 32]. This parasitic nematode directly transmitted to human *via* a faecal-oral route [accidental ingestion of its infective eggs (from contaminated water, soil or vegetables) or infective larvae (from raw/undercooked viscera of paratenic hosts)] leads to human infection [33–35]. Young children and owners of dogs are more likely to be infected due to the higher chance of contacting with infective eggs of *T. canis*. Infection with this parasite usually results in allergic (e.g. pruritus and asthma) and neurological disorders (e.g. epilepsy, idiopathic Parkinson’s disease and dementia) as well as eye diseases [36–41]. However, there are difficulties in the diagnosis and treatment of this parasitic disease [32, 42, 43], due to limited understanding of *T. canis* at molecular level.

Recent genomic and transcriptomic studies on this parasite have indicated that *Tc-aqp-1* might play important roles in the host-parasite interactions [44–46]. In our recent work [30], we have revealed the transcription profile of *Tc-aqp-1* in the different tissues of adult *T. canis*. As a logical extension, in this study, we explore the tissue expression and function of *Tc*AQP1 protein in the adult (female and male) worms of *T. canis*, using immunohistochemistry, RNA interference and nematocidal assays.

## Methods

### Parasite

Adult *T. canis* worms were collected from naturally infected dogs, which is approved by Southwest University, China, and complied with the requirements of the Ethics Procedures and Guidelines of the People’s Republic of China. Worms were washed five times in phosphate-buffered saline (PBS; pH 7.4, 37 °C) and then cultured in RPMI 1640 at 37 °C, 5% CO_2_. Worms for RNA extraction were snap-frozen in liquid nitrogen and stored at − 80 °C.

### Prokaryotic expression of recombinant C-terminal *Tc*AQP1

Total RNA was extracted from the adult worms of *T. cani*s using Trizol reagent (Invitrogen, Carlsbad, CA, USA) and reversely transcribed into the first-strand cDNA with M-MLV Reverse Transcriptase (Takara Bio, Shiga, Japan). Based on the coding sequence of *Tc-aqp-1* (GenBank: ALU85320), the nucleotide sequence coding for the C-terminal hydrophilic domain of *Tc*AQP1 (His_283_-Ala_310_) was amplified by polymerase chain reaction (PCR) using forward primer 5′-CGC GGA TCC CAC CCT TCA CCA ATT TAC ATG AA-3′ (*BamH*I restriction sites underlined) and reverse primer 5′-AAA TGC GGC CGC TTA AGC GAG GTC TGA ATT TTT-3′ (*Not*I restriction sites underlined). The PCR product was purified and inserted into pET32a vector (Takara Bio, Shiga, Japan) via *BamH*I and *Not*I restriction sites. The recombinant plasmids were amplified in *Escherichia coli* DH5α (Takara Bio, Shiga, Japan) and confirmed by DNA sequencing. BL21(DE3) *E. coli* (Takara Bio, Shiga, Japan) was transformed with the recombinant plasmids for the expression of recombinant C-terminal *Tc*AQP1. In brief, the transformed *E. coli* was cultured in Luria-Bertani broth containing 100 mg/ml ampicillin till OD_600_ = ~0.6, then induced by 1.0 mM of isopropy1-β-d-thiogalactoside at 37 °C for 4 h. Sulfate-polyacrylamide gel electrophoresis (SDS-PAGE) was used to analyse the protein expression of recombinant C-terminal *Tc*AQP1 (r*Tc*AQP1c). Expressed r*Tc*AQP1c peptide was purified using a Ni^2+^-nitrilotriacetic-acid (Ni-NTA) resin column (Sangon Biotech, Shanghai, China) and eluted using 20 mM, 50 mM, 100 mM, 250 mM or 500 mM of imidazole.

### Preparation of polyclonal antibody against r*Tc*AQP1c peptide

Pre-immune serum and polyclonal antiserum against the r*Tc*AQP1c were produced by a scientific service provider GL Biochem, Shanghai. Briefly, two New Zealand white rabbits were subcutaneously injected with the purified r*Tc*AQP1c peptide (~ 250 µg), then challenged by injecting the same amount of the antigen at 14 and 21 days following the initial injection. Rabbit anti-r*Tc*AQP1c antiserum was harvested at seven days after the last injection. The concentration and titration of the anti-r*Tc*AQP1c antibodies were estimated by bicinchoninic acid (BCA) assay and enzyme-linked immunosorbent assay (ELISA), respectively. To test the specificity of the polyclonal antibodies, western blotting was performed using the pre-immune serum (1:5000) and polyclonal anti-r*Tc*AQP1c serum (1:5000) as well as peroxidase-conjugated goat anti-rabbit IgG secondary antibody (1:5000).

### Indirect-fluorescence immunohistochemistry

Immunofluorescence analysis was performed to determine the tissue distribution of *Tc*AQP1 in the adult stage of *T. canis*. Adult worms (female and male) were fixed in 4% (w/v) paraformaldehyde/PBS overnight, then embedded in paraffin. Tissue sections (5 mm thick) prepared from the fixed worms were processed with xylene, ethanol, 3% (v/v) H_2_O_2_/PBS and 10 mM citrate buffer (pH 6.0), then blocked with 1% (v/v) bovine serum albumin (BSA)/PBS at 4 °C overnight. The blocked sections were incubated with the rabbit anti-r*Tc*AQP1c serum (1:500 in 1% BSA) at 37 °C for 4 h, fluorescein isothiocyanate (FITC)-labelled goat anti-rabbit IgG secondary antibody (1:1000 in 1%BSA) (Solarbio, Beijing, China) at 37 °C for 1 h, and 4-6-diamidino-2-phenylindole (DAPI) for 5 min. Pre-immune serum (1:500 in 1% BSA) was used as a negative control. Fluorescence signals were detected and collected using an Olympus fluorescence imaging microscope (Tokyo, Japan).

### RNA interference (RNAi)

In order to test the function of *Tc*AQP1, we conducted RNAi assay by soaking adult worms with silencing RNA targeting *Tc-aqp-1*. Small interfering RNA (siRNA; 19 nt in length) targeting *Tc-aqp-1* and negative control (non-silencing) RNA were designed using BLOCK-iT™ RNAi Designer. To check the specificity of the designed silencing RNAs (5′-GCGUGUACACUAUCUCCAA-3′) and non-silencing RNA (5′-UUCUUCGAACGUGUCACGU-3′), we manually searched these sequences against the draft genome of *T. canis* (see Zhu et al. [45]). Double-stranded RNAs with dTdT overhangs were synthesised by a scientific service provider GenePharma, Shanghai. Worms were treated with the silencing or non-silencing RNA (200 nM) in RPMI-1640 at 37 °C, 5% CO_2_ for 24 h. Nuclease-free water was used as untreated/blank control. Worm motility was checked every 6 h. The RNAi assay was conducted in triplicate, and each replicate included ≥ 10 worms.

### Quantitative real-time PCR (qRT-PCR)

After soaking for 24 h, the efficacy of gene knockdown was determined by comparing the relative mRNA levels of *Tc-aqp-*1 between the worms treated with silencing and non-silencing RNAs. qRT-PCR was performed as described previously [30] to confirm the efficiency of gene knockdown. In brief, total RNA was extracted from an individual worm (*n* = 3) and reversely transcribed. Primer set (5′-ATG CCA GTT CGA TCT CAG CC-3′ and 5′-ACG TGA ATG AGG GGC AAC TT-3′) was used to amplify *Tc-aqp-*1. Small subunit ribosomal RNA (18S rRNA) (5′-AAT TGT TGG TCT TCA ACG AGG A-3′ and 5′-AAA GGG CAG GGA CGT AGT CA A-3′) was used as the internal standard. The relative transcriptional levels of *Tc-aqp-*1 in the worms treated with silencing and non-silencing RNA were calculated using the 2^−ΔΔCt^ method, with reference to that in untreated worms. qRT-PCR was performed in triplicate. Statistical analyses (Student’s t-test) were performed using Prism 7 (GraphPad, La Jolla, USA).

### Nematocidal assay

To understand whether *Tc*AQP1 plays a role in drug uptake, we tested the nematocidal activity of albendazole on the adult worms *in vitro*, immediately following the RNAi assay. The worms (*n* = 10) soaked with silencing, non-silencing RNA or nuclease-free water were transferred to fresh RPMI-1640 medium supplemented with or without 0.2 mg/ml of albendazole, and incubated at 37 °C, 5% CO_2_ for 2 h. Worm motility was checked for 5 min every 30 min, and the number of worms survived from the albendazole treatment was counted. The nematocidal assay was conducted in triplicate. Statistical analyses (Student’s t-test) were performed using Prism 7 (GraphPad, La Jolla, USA).

## Results

### Specific polyclonal antibody against r*Tc*AQP1c peptide

**Fig. 1:**
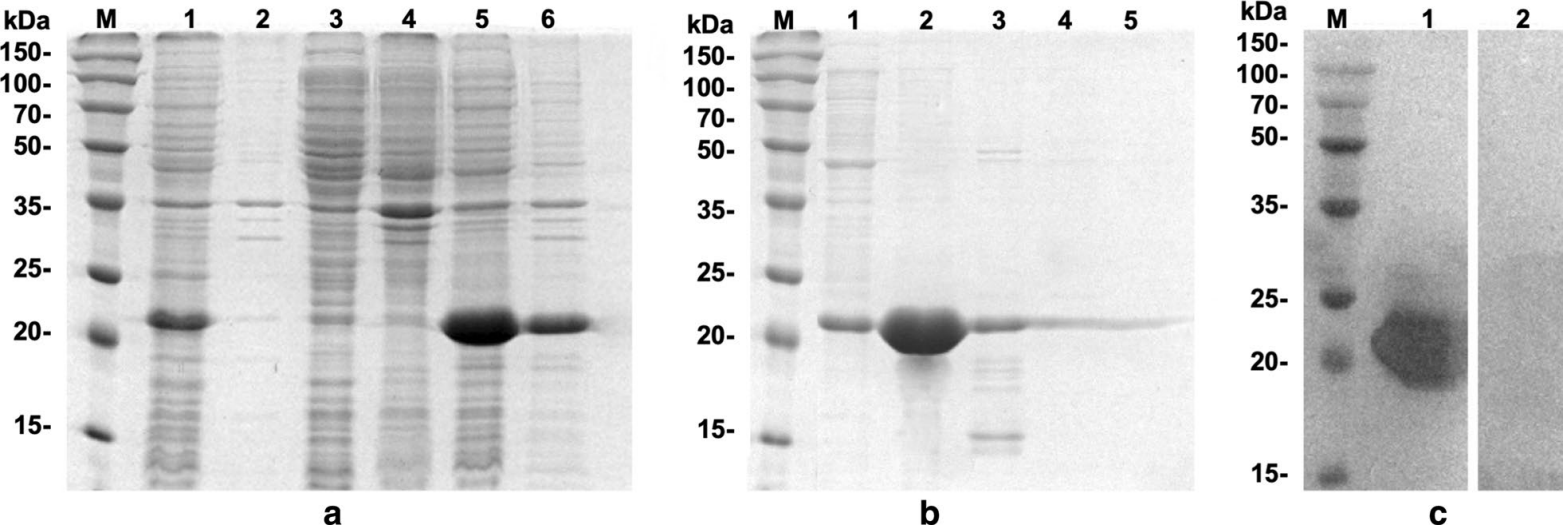
Expression, purification and western blotting of recombinant *Tc*AQP1c peptide. **a** SDS-PAGE analysis of the prokaryotic expression of recombinant *Tc*AQP1c. Lane M: protein molecular weight maker; Lane 1: supernatant expression of induced empty pET32a; Lane 2: insoluble expression of induced empty pET32a; Lane 3: supernatant expression of non-induced recombinant pET32a plasmids; Lane 4: insoluble expression of non-induced recombinant pET32a plasmids; Lane 5: supernatant expression of induced recombinant pET32a plasmids; Lane 6: insoluble expression of induced recombinant pET32a plasmids. **b** Purification of recombinant *Tc*AQP1c peptide. Lane M: protein molecular weight maker; Lanes 1–5: elution using 20 mM, 50 mM, 100 mM, 250 mM or 500 mM of imidazole, respectively. **c** Western blot analysis of recombinant *Tc*AQP1c peptide. Lane M: protein molecular weight maker; Lane 1: polyclonal antibody against the recombinant *Tc*AQP1c peptide; Lane 2: pre-immune serum

The C-terminal hydrophilic domain of *Tc*AQP1 was expressed in BL21 (DE3) cells as a 6× His-GST fusion protein (r*Tc*AQP1c) (Fig. 1a). The fusion protein was enriched using a Ni-NTA chromatography column and eluted with 50 mM of imidazole, resulting in the purified r*Tc*AQP1c peptide (Fig. 1b). Polyclonal antibody against the r*Tc*AQP1c peptide (anti-r*Tc*AQP1c) was produced, with protein concentration estimated at 0.86 mg/ml and titration at > 1:64,000. Western blot analysis showed a specific binding activity of the polyclonal antibody and the r*Tc*AQP1c peptide (Fig. 1c). No obvious binding was observed between the pre-immune serum and the r*Tc*AQP1c peptide (Fig. 1c).

### Tissue distribution of *Tc*AQP1 in adult worms

**Fig. 2:**
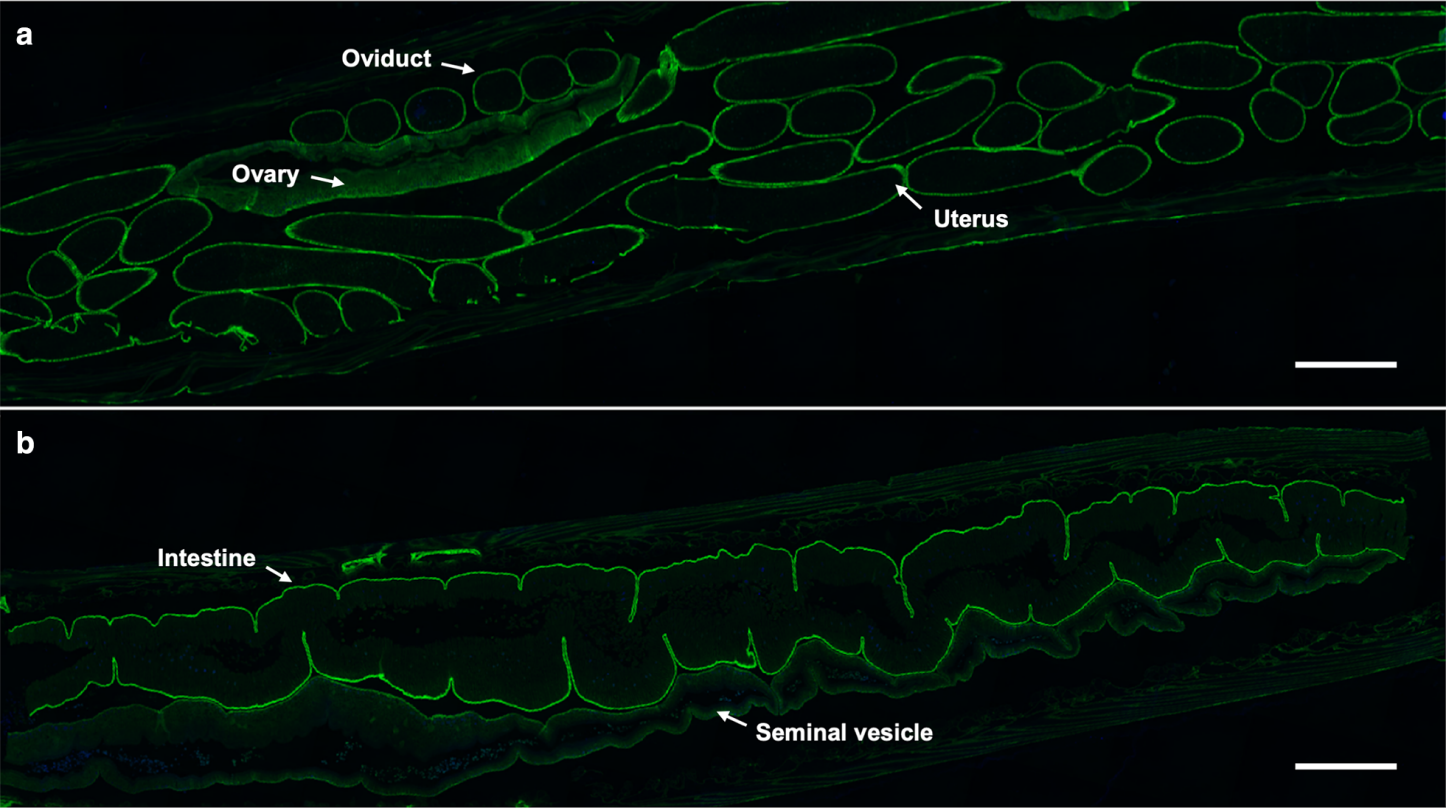
Immunofluorescence microscopy of the tissue distribution of *Tc*AQP1 in the adult stage of *Toxocara canis*. Tissue distribution of *Tc*AQP1 in female (**a**) and male (**b**) adult stage of *T. canis*. Reproductive organs (ovary, oviduct, uterus and seminal vesicle) and intestine are indicated by white arrows. *Scale-bars*: 1000 μm

Having produced specific polyclonal antibody against r*Tc*AQP1c, we sought to study the tissue distribution of native *Tc*AQP1 in the adult stage (female and male) of *T. canis*. Using the anti-r*Tc*AQP1c, tissue distribution of native *Tc*AQP1 was indicated by indirect immunofluorescence signals (Fig. 2). Specifically, predominant fluorescence signals were detected from the reproductive tract (ovary, oviduct, uterus) of female worm (Fig. 2a), and from the seminal vesical and intestine of male adult worm (Fig. 2b). Particularly, *Tc*AQP1 was localised in the epithelial linings of reproductive organs and intestinal basolateral membrane (Fig. 2a, b). No signal was observed in the control assay using the pre-immune serum.

### Gene knockdown of *Tc*-*aqp-1*

After culturing in RPMI-1640 medium for 24 h, the treated and untreated adult worms did not show a significant reduction of motility. No obvious difference in motility was seen between the worms soaked with silencing and non-silencing RNAs.

**Fig. 3:**
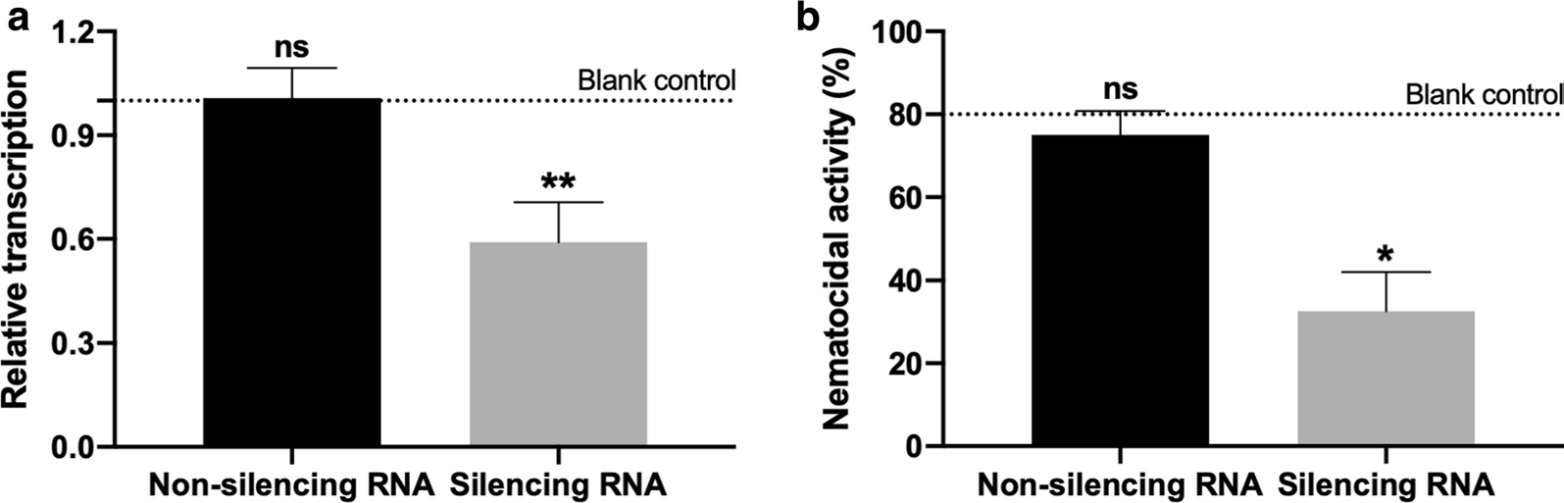
Gene knockdown and nematocidal analyses. **a** Relative transcription of *Tc-aqp-1* in adult worms soaked with non-silencing and silencing RNAs are determined with reference to blank control. **b** Nematocidal activity of albendazole on non-silenced and silenced worms are compared with respect to blank control. Error bar indicates a standard deviation (SD). Statistical significance (Student’s t-test) is indicated with asterisks (*P < 0.05, **P < 0.01)

To determine whether the *Tc*-*aqp-1* gene was efficiently silenced, we compared the mRNA level of *Tc*-*aqp-1* between treated and untreated adult worms after soaking for 24 h. We found that the siRNA (5′-GCGUGUACACUAUCUCCAA-3′) targeting the open reading frame of *Tc*-*aqp-1* (G245-A263) significantly reduced the transcription of *Tc*-*aqp-1* in adult worms, with respect to that in untreated worms (*t*_(6)_ = 5.76, *P *= 0.001; Fig. 3a). No significant difference was found in the mRNA levels of *Tc*-*aqp-1* between non-silencing RNA-treated and untreated worms (*t*_(6)_ = 0.02, *P *= 0.098) (Fig. 3a). No obvious phenotypic change (e.g. motility) was observed in treated and untreated worms.

### Silenced *Tc*-*aqp-1* compromised nematocidal activity of albendazole

As the mRNA level of *Tc*-*aqp-1* has been efficiently reduced in the adult worms, we sought to test whether this gene knockdown would affect the function of *Tc*AQP1 (possibly drug transport or uptake). Treatment with 0.2 mg/ml of albendazole for 2 h resulted in a lethal phenotype (no motion for 5 min) for ~40% of the silenced worms, compared with ~ 70% of the non-silenced worms (Fig. 3b). A significant difference (*t*_(6)_ = 5.91, *P *= 0.098) was found in the nematocidal activity of albendazole between the silenced and non-silenced worms *in vitro*. No significant difference was found between non-silenced and untreated worms (*t*_(6)_ = 1.00, *P *= 0.36).

## Discussion

In this study, we determined the tissue expression of *Tc*AQP1, which is localised on the reproductive and intestinal tracts in the adult (female and male) worms of *T. canis*. Gene knockdown of the protein-coding gene *Tc-aqp-1* resulted in a significant reduction of mRNA level, and consequently, compromised the nematocidal activity of albendazole *in vitro*.

The protein expression of *Tc*AQP1 in the epithelial linings of reproductive tract indicates biological roles in gametogenesis in adult *T. canis*. Similar tissue (i.e. testes and ovary) expression has been reported for AQP1 in *Fasciola gigantica* [14], which has been proposed to be associated with the production of seminal fluids [47]. However, in *C. elegans*, AQP3, rather than AQP1, has been reported to express predominantly in seminal vesicle and vas deferens [4]. The discrepancy between the free-living *C. elegans* and the parasitic *T. canis* might suggest evolutionary divergence, which clearly warrants further testing as there is a lack of information about other AQPs in the latter species. A transcriptomic study of *Tc-aqp-1* or a proteomic study of *Tc*AQP1 across the key developmental stages of *T. canis* would provide insights into the functional roles of the gene or protein in this parasite.

The predominant distribution of *Tc*AQP1 in intestinal membrane highlights its functions in nutrient, solute transport or osmoregulation. The functional roles of AQPs in transporting water, glycerol and urea have been studied in several platyhelminths, such as *F. gigantica*, *Opisthorchis viverrini* and *S. mansoni* [14, 18, 20]. In these worms, AQPs have been frequently shown in the tegument cells [14, 20, 48]. Although nematodes do not have tegument, which have evolved to possess the specialised coat (cuticle), the functional roles of intestinal AQPs in nematodes should be similar to the tegument AQPs in trematodes as both of them are important organs known for nutrient absorption. This proposal can be somewhat supported by the predominant gene transcription of *Tc-aqp-1* and protein expression of *Tc*AQP1 in the intestine of adult *T. canis* [30]. Interestingly, *Tc*AQP1 was localised specifically on the intestinal basolateral membrane, which is consistent with the tissue expression of AQP1 in *C. elegans* [4]. This specific distribution suggests an adaptation to the chronic hypertonic stress in the intestine of the host, because it is known to transport intestinal glycerol into the pseudocoelomic cavity to maintain all non-glycerol-producing cells [49, 50]. This hypothesis should be tested by the use of genetic tools, such as RNAi and CRISPR/CAS9, which must be based on a well-established *in vitro* culture system.

Importantly, we silenced the gene *Tc-aqp-1* using an established soaking approach for the adult stage of *T. canis in vitro* [51]. Although the effective gene knockdown significantly resulted in reduced mRNA level of *Tc-aqp-1*, it did not lead to a lethal or suppressed phenotype. This might be explained by the short term (24 h) of soaking with silencing RNA, which limited us to explore the role of *Tc-aqp-1* in reproductive activity but happened to facilitate us to test the roles of *Tc-aqp-1* in drug uptake. The roles of AQPs in drug uptake have been investigated in *S. mansoni*, *Leishmania* spp. and *T. brucei* [20–23], but little is known about this area in nematodes. Here, we found that the silenced (partially) *Tc-aqp-1* compromised the nematocidal activity of albendazole (a broad-spectrum anthelmintic commonly used for the treatment of toxocariasis), suggesting that *Tc*AQP1 might play a role in the uptake of this anthelmintic. However, this suggestion warrants further testing considering the complex pharmacokinetics of albendazole (e.g. inhibiting polymerization of β-tubulin and/or inducing degenerative alterations) [52]. A comparative analysis of the concentrations of albendazole among the tissues (intestine, reproductive organs and musculature) in the treated worms would provide a better understanding whether *Tc*AQP1 play a role in drug uptake.

Taken together, *Tc*AQP1 might represent a novel anthelmintic target. First, the high transcriptional level of *Tc-aqp-1* in the infective larvae of *T. canis* suggests its roles in host-parasite interaction [30, 44, 45]. Second, the predominant expression of *Tc*AQP1 in the intestine of adult *T. canis* indicates the crucial roles of this protein in parasite survival, which could be easily targeted. Although a previous immunisation with *S. mansoni* AQP1 failed to reduce worm burden and liver pathology in a murine model [53], it is still worthwhile to evaluate the potentiality of *Tc*AQP1 as a novel drug target [54, 55], since it might play important roles in reproductive processes of *T. canis*. Clearly, further studies are warranted to have a better understanding of *Tc-aqp-1* or *Tc*AQP1 in other stages of this parasitic nematodes.

## Conclusions

Here, we reported for the first time the tissue distribution of *Tc*AQP1 in the adults of *T. canis*, which might be associated with reproduction, nutrient absorption and/or osmoregulation. Gene knockdown of *Tc-aqp-1* compromised the nematocidal activity of albendazole *in vitro*, suggesting a role of *Tc*AQP1 in drug uptake. A better understanding of this membrane channel protein would contribute to the discovery of novel intervention strategy.

## Data Availability

The datasets supporting the conclusions of this article are included within the article.

## Abbreviations

*Tc-aqp-1*: aquaporin 1 gene of *Toxocara canis*
*Tc*AQP1: aquaporin 1 protein of *Toxocara canis*
r*Tc*AQP1c: recombinant the C-terminal hydrophilic domain of *Tc*AQP1
RNAi: RNA interference
qRT-PCR: quantitative real-time polymerase chain reaction
